# Vascular Alterations Preceding Arterial Wall Thickening in Overweight and Obese Children

**DOI:** 10.3390/jcm11123520

**Published:** 2022-06-19

**Authors:** Sung-Ai Kim, Kyung Hee Park, Sarah Woo, Yoon Myung Kim, Hyun Jung Lim, Woo-Jung Park

**Affiliations:** 1 Division of Cardiology, Department of Internal Medicine, Hallym Sacred Heart Hospital, Hallym University College of Medicine, Anyang 14068, Korea; cathpark@hallym.or.kr; 2 Department of Family Medicine, Hallym Sacred Heart Hospital, Hallym University College of Medicine, Anyang 14068, Korea; beloved@hallym.or.kr; 3 Department of Medical Science, Hallym University College of Medicine, Anyang 14068, Korea; hjejcross@naver.com; 4 Department of Sports Industry Studies, Yonsei University International Campus, Incheon 21983, Korea; yoonkim@yonsei.ac.kr; 5 Department of Medical Nutrition, Kyung Hee University, Yongin 17104, Korea; hjlim@khu.ac.kr

**Keywords:** obesity, childhood, vascular, stiffness

## Abstract

Background: Childhood obesity is linked to adverse cardiovascular outcomes in adulthood. This study aimed to assess the impact of childhood obesity on the vasculature and to investigate whether vascular alteration precedes arterial wall thickening in childhood. Methods: A total of 295 overweight (body mass index [BMI] 85th to 95th percentile, *n* = 30) and obese (BMI ≥ 95th percentile, *n* = 234) children aged 7–17 years and 31 normal-weight controls with similar age and gender were prospectively recruited. We assessed anthropometric data and laboratory findings, and measured the carotid intima–media thickness (IMT), carotid artery (CA) diameter, M-mode-derived arterial stiffness indices, and velocity vector imaging parameters, including the CA area, fractional area change, circumferential strain, and circumferential strain rate (SR). Results: The mean ± standard deviation age of the participants was 10.8 ± 2.1 years; 172 (58%) children were male. Regarding structural properties, there was no difference in the IMT between the three groups. The CA diameter was significantly increased in obese children, whereas the CA area showed a significant increase beginning in the overweight stage. Regarding functional properties, contrary to β stiffness and Young’s elastic modulus, which were not different between the three groups, the circumferential SR showed a significant decrease beginning in the overweight stage and was independently associated with BMI z-scores after adjusting for covariates. Conclusion: We have demonstrated that arterial stiffening and arterial enlargement precede arterial wall thickening, and that these vascular alterations begin at the overweight stage in middle childhood or early adolescence.

## 1. Introduction

Obesity leads to increased arterial stiffening and increased intima–media thickness (IMT), both of which have been linked to a pathological cascade of cardiovascular (CV) diseases [1]. Recently, childhood obesity has become a public health problem worldwide; its prevalence among children has been increasing due to a sedentary lifestyle and fast-food consumption [2]. As obese children are not only more likely to become obese adults but also have an increased risk of developing hypertension, dyslipidemia, type 2 diabetes mellitus, and future CV disease, childhood obesity will eventually be one of the most serious global health issues [3,4]. Large epidemiological studies have reported that childhood obesity is linked to adverse vascular alterations in adulthood [5,6,7]. Although the association between obesity and vascular alteration has been extensively investigated in adulthood, limited data exist on its impact on the vasculature in childhood. As surrogate markers of vascular alteration, both the IMT and arterial stiffness are known as independent predictors of future CV morbidity and mortality in adults, respectively [8]. However, in childhood, previous studies regarding the impact of obesity on IMT and arterial stiffness have yielded conflicting results, and not all studies have shown higher IMT and arterial stiffness in obese children [9,10,11,12,13,14,15]. Using the velocity vector imaging (VVI) technique to assess instantaneous vascular deformation, we previously quantified vascular alterations with aging [16] and demonstrated that the carotid artery (CA) could undergo functional alteration before the IMT increases in patients with hypertension [17]. In this study, we assessed vascular alterations using VVI in overweight and obese children and compared them to normal-weight children and sought to determine whether vascular alterations could be observed before arterial wall thickening in overweight and obese children.

## 2. Materials and Methods

### 2.1. Study Subjects

A total of 295 overweight (*n* = 30) and obese (*n* = 234) children aged 7–17 years (154 boys and 110 girls) and 31 sex- and age-matched normal-weight controls were prospectively recruited for the Intervention for Childhood and Adolescents Obesity via Activity and Nutrition (ICAAN) study through newspapers, broadcasts, posters, websites, and other social networking services. Overweight was defined as a BMI ≥ 85th percentile and <95th percentile for age- and sex-specific BMI according to the 2007 Korean National Growth Charts, while obesity was defined as a BMI ≥ 95th percentile for age and sex or a BMI ≥ 25 kg/m^2^ [18,19]. This study was conducted according to the guidelines of the Declaration of Helsinki, and the study protocol was approved by the local ethics committee. Written informed consent was obtained from all participants and their parents or caregivers.

### 2.2. Anthropometric Data

Body weight was measured after a 10 h fast and voiding, with the participants barefoot and wearing indoor and lightweight clothing. Height was measured by a stadiometer (DS-103, DongSahn Jenix, Seoul, Korea) while the participants were barefoot. BMI was calculated (weight [kg]/height [m]^2^) and converted into percentiles and z-scores based on the age- and sex-specific BMI of the 2007 Korean National Growth Charts [18].

### 2.3. Laboratory Test

Venous blood samples were obtained after 12 hours of fasting to determine the fasting plasma glucose (FPG), fasting plasma insulin, high-density lipoprotein cholesterol (HDL-C), low-density lipoprotein cholesterol (LDL-C), triglyceride (TG), aspartate aminotransferase (AST), alanine aminotransferase (ALT), and high-sensitivity C-reactive protein (hsCRP). FPG was measured using ultraviolet assay with hexokinase (Cobas 8000 C702, Roche, Mannheim, Germany). Insulin was measured using electrochemiluminescence immunoassay (Cobas 8000 e802, Roche, Mannheim, Germany). HDL-C and LDL-C were measured using a homogeneous enzymatic colorimetric test (Cobas 8000 C702, Roche, Mannheim, Germany). TG, AST, and ALT were measured using enzymatic assay (Cobas 8000 C702, Roche, Mannheim, Germany). hsCRP was measured using turbidimetric immunoassay (Cobas 8000 C702, Roche, Mannheim, Germany). The homeostasis model assessment for insulin resistance (HOMA-IR) was used to determine insulin sensitivity, and was calculated using the following formula: (FPG Level (mg/dL) × Fasting Plasma Insulin Level (uU/mL))/405. Adiponectin was measured using enzyme-linked immunosorbent assay (VersaMax ELISA Microplate Reader, Molecular Devices, San Jose, CA, USA).

### 2.4. Carotid Ultrasound and Vascular Parameters

Carotid ultrasound studies were performed by a single registered vascular technologist (S.H.P) who was blinded to the subject group assignment using a high-resolution B-mode ultrasound (Acuson Sequoia 512, Siemens Acuson, Mountain View, CA, USA) equipped with an 8 MHz linear-array transducer. Data were stored as digital cineloops for subsequent offline analysis, and a single experienced reader (S.A.K.) who was blinded to the subject’s clinical status performed all measurements. The mean IMT was calculated as the average of three consecutive manual measurements at the far wall of the CA 1 cm proximal to the carotid bulb of both common carotid arteries (CCA) from leading edge (lumen–intima) to leading edge (media-adventitia) during end diastole. The average end-diastolic diameter (Dd, CA diameter) and peak systolic internal diameters (Ds) were assessed in three cycles as the distance between the intima–lumen interface at the near wall and the lumen–intima interface at the far wall of both CCAs (1 cm proximal to the beginning of the carotid bulb). The M-mode-derived CA stiffness indices were derived according to the following formula: β stiffness was determined as ln (Ps/Pd)/((Ds − Dd)/Dd) [20], where Ps and Pd are systolic and diastolic blood pressures. Young’s elastic modulus (YEM) in 10^2^ × kPa/mm was calculated as [[Ps − Pd]/(Ds − Dd)] × (Dd/cIMT) [21]. Transverse images of both CCAs (1 cm proximal to the carotid bulb) were stored using acoustic capture for offline analysis with the VVI workstation (Syngo^®^, US Workplace, Siemens, Mountain View, CA, USA). VVI fundamentally uses a two-dimensional speckle-tracking method from which the blood–tissue border was traced manually over one frame of a cineloop and wherein the ultrasonic speckles automatically tracked vessel wall motion by dividing it into six segments. 

VVI provides instantaneous quantitative measurements of vessel deformation throughout the cardiac cycle, including circumferential vessel area, fractional area change (FAC), circumferential strain and strain rate (SR). CA area was defined as minimal vessel area during the cardiac cycle and FAC was calculated by measuring the percent changes of the CA area [(maximal area) − (minimal area)/(minimal area)] × 100 (%). Circumferential strain (ε) represents the percent change (%) in length along the circumferential axis of CA and circumferential SR represents the temporal derivative of strain and describes the temporal change in strain (dε/dt, 1/s), producing a positive value in systole and a negative value in diastole. All CA measurements on both sides were averaged to obtain the mean values.

### 2.5. Statistical Analysis

Data are expressed as the mean ± standard deviation (SD) or as percentages. We compared the means of each continuous variable in the subject groups by one-way factorial analysis of variance with the post hoc test (Tukey). Analysis of covariance using the Bonferroni post hoc test was used to test the differences in the vascular parameters between the three groups while adjusting for covariates, such as age, sex, height, mean blood pressure, low-density lipoprotein (LDL)-cholesterol level, and homeostasis model assessment for insulin resistance (HOMA-IR). Multiple linear regression fit of the circumferential SR on the BMI z-score was generated by considering the effects of other covariates. All statistical analyses were performed using SPSS 24.0 (IBM Corp., Chicago, IL, USA) an open-source statistical package R version 3.6.3 (R Project for Statistical Computing, Vienna, Austria). Statistical significance was defined as *p* < 0.05.

## 3. Results

Overall, the mean ± SD age of all participants was 10.8 ± 2.1 years; 172 (58%) children were male. Table 1 presents the baseline characteristics of the study population and statistical differences between the groups.

Obese children had higher blood pressures, HOMA-IR, hsCRP, and LDL cholesterol levels and lower serum adiponectin levels than overweight and normal-weight children. Meanwhile, overweight children had higher levels of HOMA-IR compared to normal-weight children, although there were no differences in other variables between the groups.

In the structural evaluation of the CA (Table 2), there was no difference in IMT among the three groups (*p* > 0.05).

The CA diameter in obese children was distinctly larger than that in normal-weight and overweight children, and there was no difference in the CA diameter between normal-weight and overweight children. Meanwhile, the CA area began to increase from the overweight stage. The CA of overweight children was significantly larger than that of normal-weight children but did not differ from obese children. Regarding the functional parameters of vascular elastic properties, M-mode-derived stiffness indices, such as β stiffness and YEM, were not significantly different among the three groups (*p* > 0.05). Meanwhile, the circumferential SR showed a significant decrease from the overweight stage (0.68 ± 0.24 1/s in normal-weight vs. 0.51 ± 0.21 1/s in overweight children, *p* = 0.002). 

**Figure 1:**
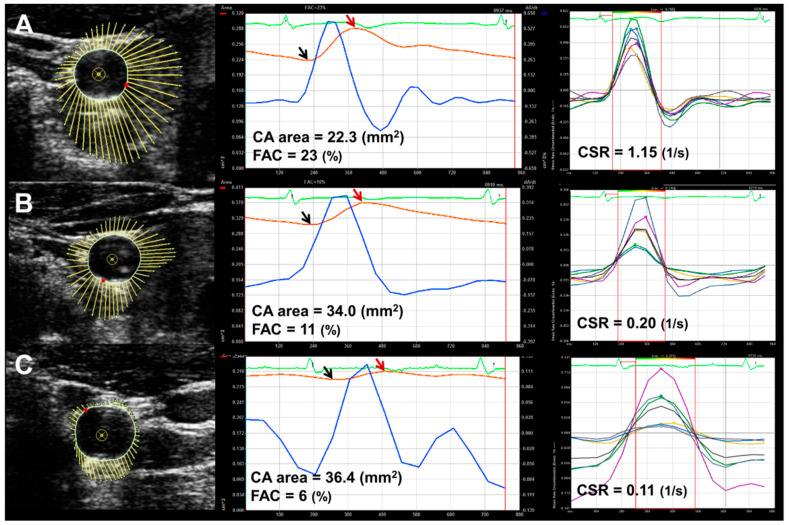
Velocity vector imaging analysis in normal-weight (**A**), overweight (**B**) and obese children (**C**). The red arrow points to maximal CA area and the black arrow points to minimal CA area during the cardiac cycle. CA area refers to minimal CA area (black arrow) and FAC was calculated by measuring the percent changes of the CA area [(maximal area) − (minimal area)/(minimal area)] × 100 (%). CSR refers to average value of peak CSRs of six segments during the systole. Compared to normal-weight children (**A**), overweight (**B**) and obese (**C**) children show an increase in CA area and a decrease in FAC and CSR. CA, carotid artery; FAC, fractional area change; CSR, circumferential strain rate.

Figure 1 shows an example of VVI analysis in normal-weight (A), overweight (B) and obese children (C), respectively. As the BMI level increases, CA area gradually increases and both FAC and circumferential SR decrease in comparison with those of normal-weight children.

**Figure 2:**
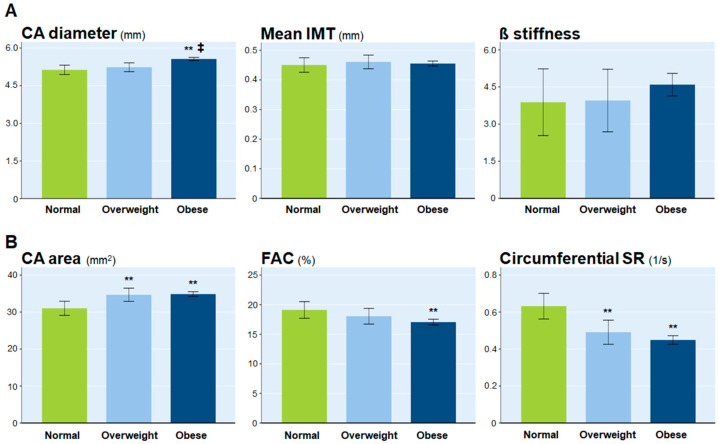
Comparison of M-mode-derived parameters and VVI parameters between the groups after adjusting for covariates including age, sex, height, mean blood pressure, LDL cholesterol and HOMA-IR. (**A**) M-mode-derived parameters; (**B**) VVI parameters. ** vs. normal, <0.01; ‡ vs. overweight < 0.01. CA, carotid artery; IMT, intima–media thickness; FAC, fractional area change; SR, strain rate; VVI, velocity vector imaging.

In addition, when we compared the M-mode-derived parameters and VVI parameters between the groups after adjusting for age, sex, height, mean blood pressure, LDL cholesterol level, and HOMA-IR, respectively (Figure 2), the CA area and circumferential SR still showed significant changes beginning in the overweight stage in middle childhood or early adolescence. 

**Figure 3:**
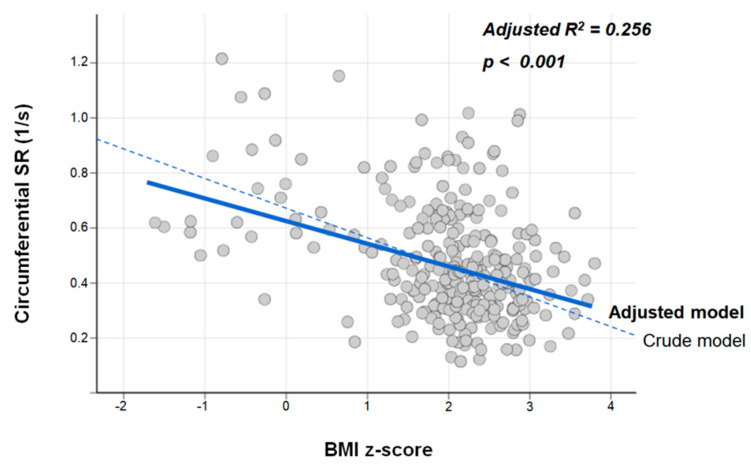
Multiple linear regression fit of the circumferential SR on the BMI z-score. SR, strain rate; BMI, body mass index.

Additionally, the circumferential SR showed a negative association with the BMI z-score, a linear estimate of obesity; this association remained significant after adjustment of covariates in the multiple linear regression analysis (adjusted R^2^ = 0.256, *p* < 0.001, Figure 3). 

## 4. Discussion

In this study, we demonstrated that even in childhood, arterial stiffening and enlargement, which occur in the overweight stage, precede arterial wall thickening. The circumferential SR, as a sensitive marker of arterial stiffness, significantly decreased in the early stages of obesity and showed a negative linear association with BMI z-score even after adjusting for covariates. Our results emphasize that interventions against childhood obesity should be initiated early to prevent the induction and irreversible progression of obesity-induced vascular complications.

The IMT is an established structural marker of subclinical arteriosclerosis [9,22]. Many studies in adults have demonstrated that obesity is independently associated with an increase in IMT [23,24,25]. Moreover, large longitudinal cohort studies have shown that childhood adiposity correlates with an increase in IMT later in adulthood [26,27]. However, some studies have reported the lack of association between BMI and IMT in childhood [9,11,28,29,30,31,32,33]. In middle childhood (mean age, 10 years; <12 years), BMI is not associated with IMT in obese and normal-weight children [11,28,29,30], whereas in adolescents (mean age, >12 years), BMI is positively associated with IMT [31,32,33]. This suggests that arterial wall damage begins later in childhood or in adolescence. Our results are also in line with those of previous reports of children aged 8–12 years that did not show a difference in the IMT between obese and normal-weight subjects [11,28,29,30]. Moreover, this study including overweight children, who were greater in number than obese children, showed that the IMTs in overweight and obese children were not different to those of normal-weight children, which suggests that the thickening of the arterial wall is limited by time in childhood obesity. In contrast, several studies with similar degree of obesity, age, and gender to this population have reported an increase in IMT in obese children compared with lean children [9,12,34]. These conflicting reports could be due to the differences in exposure duration to obesity, sample size, and racial and/or ethnic characteristics between studies [35,36]. Another possible explanation is that BMI or adiposity itself has no direct association with IMT, and that its effects are instead manifested through CV risk factors, such as age, hypertension, type 2 diabetes mellitus, and other metabolic complications [36,37]. It is also fundamentally linked to exposure duration to obesity.

In this study, we verified that arterial stiffening precedes wall thickening, and that arterial damage begins at the overweight stage in middle childhood or early adolescence. Although longitudinal processes linking obesity to vascular alteration are not fully understood, it is known that long-term exposure to hemodynamic stimuli and metabolic disturbance caused by obesity augments the arterial impedance and afterload of the heart, which eventually leads to an increase in CV morbidity and mortality [38]. In this study, overweight children showed higher insulin resistance than normal-weight children despite the comparable blood pressures and laboratory findings. Although the role of this metabolic disturbance in the process of arteriosclerosis remains unclear, abnormal glucose metabolism is associated with the accumulation of advanced end-glycation products that lead to arterial stiffening [39]. To assess arterial stiffness, we measured the instantaneous vessel deformational parameters (FAC, circumferential strain, and circumferential SR) using VVI as well as conventional M-mode-derived elastic moduli as β stiffness and YEM. 

Although the M-mode-derived stiffness indices did not show any difference among the three groups, and the FAC and circumferential strain of overweight children were not significantly different from those of normal-weight children, only circumferential SR has proven its ability to independently confirm early vascular damage that began at the overweight stage and it showed a negative linear association with BMI z-score even after adjusting for covariates. M-mode derived stiffness indices as β-stiffness and YEM are calculated from CA diameter and blood pressures measured at peripheral artery. When interpreting the results, we should consider the facts that pulse pressure amplification may differ with obesity [40] and there is a time difference between the measurements of blood pressures and arterial diameter in pressure-related variables. These limitations may have affected the findings in this study.

Considering that the circumferential SR is the time rate of instantaneous circumferential deformation, it would have been a suitable indicator to reflect subtle vascular changes in these relatively healthy children, although the exact mechanisms cannot be clarified. Additionally, our results revealed the limitations of the M-mode-derived stiffness indices and the superiority of a two-dimensional approach by speckle tracking that enables instantaneous vessel wall motion in the entire circumference of vessel wall.

As a marker of structural change, we demonstrated that vessel area increases as obesity progresses, which is consistent with existing pediatric studies that reported that arterial diameters increase with obesity [41,42]. An increase in arterial diameter might be attributed to the fatiguing effects of tensile stress, which lead to fracture of load-bearing elastin fibers [43,44]. In this study, the CA area, measured by a two-dimensional approach, showed a significant increase from the overweight stage. These results reveal that arterial enlargement and stiffening occur before IMT progression and begin in the overweight stage in childhood. Considering the four large longitudinal cohort studies wherein people who were overweight or obese as children but were non-obese as adults had similar CV outcomes to those of people who were never obese [7], it is plausible that these vascular alterations in childhood are reversible and preventable through early intervention against obesity.

Our investigation has several limitations, including its cross-sectional observational study design, which does not allow causal or temporal inferences. A narrow age range in this population may limit generalizability to later adolescence, wherein the influence of pubertal development, with changes in body shape and adiposity, cannot be overlooked. It is unclear how much the duration of exposure to obesity might affect the degree of structural and functional vascular alterations in this population.

## 5. Conclusions

In conclusion, we demonstrated that vascular alterations such as arterial enlargement and arterial stiffening, represented by low circumferential SR, can occur before IMT progression even in the overweight stage in middle childhood or early adolescence. These results emphasize the importance of maintaining a normal body weight in childhood and the necessity of early intervention against childhood obesity to minimize the development of CV disease in adulthood.

## Figures and Tables

**Table 1:** Baseline characteristics.

	Normal Weight	Over Weight	Obese	*p* Value
Age, year	10.5 ± 2.4	10.5 ± 2.0	10.8 ± 2.1	0.685
Male, %	58	53	59	0.840
Height, cm	143 ± 13	145 ± 10	152 ± 12 **^,^^‡^	<0.001
Weight, kg	38.2 ± 11.8	49.9 ± 10.2 **	68.7 ± 18.1 **^,^^‡^	<0.001
BMI, kg/m^2^	18.0 ± 2.4	23.3 ± 1.8 **	28.9 ± 4.1 **^,^^‡^	<0.001
BMI z-score	−0.13 ± 0.76	1.45 ± 0.17 **	2.38 ± 0.44 **^,^^‡^	<0.001
WC, cm	60.0 ± 7.3	74.7 ± 7.2 **	89.3 ± 10.6 **^,^^‡^	<0.001
HC, cm	78.3 ± 7.9	87.0 ± 6.7 **	98.3 ± 10.4 **^,^^‡^	<0.001
Systolic BP, mmHg	104 ± 11	107 ± 8.7	114 ± 9.6 **^,^^‡^	<0.001
Diastolic BP, mmHg	60 ± 6.0	63 ± 6.0	65 ± 8.0 **	0.003
Mean BP, mmHg	75 ± 6.8	78 ± 5.8	82 ± 7.5 **^,^^†^	<0.001
Heart rate, bpm	80 ± 11	79 ± 10	80 ± 12	0.909
Hemoglobin, mg/dL	13.9 ± 0.9	13.9 ± 0.8	13.7 ± 0.8	0.477
Creatinine, mg/dL	0.55 ± 0.13	0.53 ± 0.06	0.54 ± 0.10	0.729
Glucose, mg/dL	82 ± 10	86 ± 9.9	88 ± 7.6 **	0.001
AST, IU/L	27 ± 23	23 ± 8.5	27 ± 18	0.446
ALT, IU/L	16 ± 18	21 ± 21	37 ± 40 *	0.003
HOMA-IR	1.35 ± 0.75	2.89 ± 1.80 **	4.80 ± 2.79 **^,^^‡^	<0.001
hsCRP, mg/dL	0.47 ± 0.31	0.94 ± 0.83	2.17 ± 2.11 **^,^^‡^	<0.001
TG, mg/dL	78 ± 38	104 ± 51	114 ± 56 **	0.002
HDL-C, mg/dL	60 ± 13	56 ± 14	49 ± 11 **^,^^‡^	<0001
LDL-C, mg/dL	94 ± 20	105 ± 23	110 ± 25 **	0.003
Adiponectin, ug/mL	10.2 ± 4.0	9.4 ± 4.1	8.4 ± 3.2 **	0.012

* vs. normal < 0.05; ** vs. normal, <0.01; ^†^ vs. overweight < 0.05; ^‡^ vs. overweight < 0.01. BMI, body mass index; WC, waist circumference; HC, hip circumference; BP, blood pressure; AST, aspartate aminotransferase; ALT, alanine aminotransferase; HOMA-IR, homeostasis model assessment for insulin resistance; hsCRP, high-sensitivity C-reactive protein; TG, triglyceride; HDL-C, high-density lipoprotein cholesterol; LDL-C, low-density lipoprotein cholesterol.

**Table 2:** Structural and functional characteristics of the carotid artery.

	Normal Weight	Over Weight	Obese	*p* Value
M-mode-derived parameters				
Mean IMT, mm	0.44 ± 0.04	0.45 ± 0.05	0.45 ± 0.06	0.277
CA diameter, mm	5.05 ± 0.49	5.16 ± 0.51	5.58 ± 0.53 **,^‡^	<0.001
ß stiffness	4.15 ± 1.30	4.13 ± 1.77	4.53 ± 3.77	0.735
YEM, 10^2^ × kPa/mm	5.45 ± 1.65	5.51 ± 2.44	6.34 ± 5.05 ^‡^	0.443
VVI parameters				
CA area, mm^2^	30.3 ± 5.0	34.0 ± 5.1 *	35.0 ± 5.6 **	<0.001
FAC, %	19.1 ± 4.2	17.9 ± 3.3	17.0 ± 3.6 **	0.007
Circumferential strain, %	6.50 ± 2.45	5.74 ± 1.64	5.67 ± 1.94	0.090
Circumferential SR, 1/s	0.68 ± 0.24	0.51 ± 0.21 **	0.44 ± 0.18 **	<0.001

* vs. normal < 0.05; ** vs. normal, <0.01; ^‡^ vs. overweight < 0.01. IMT, intima–media thickness; CA, carotid artery; YEM, Young’s elastic modulus; VVI, velocity vector imaging; FAC, fractional area change; SR, strain rate.

## Data Availability

The data presented in this study are available on request from the corresponding authors. The data are not publicly available due to privacy concerns.
